# Model for compassion fatigue onset in cancer care nurses: focusing on patient traumatic events and nurses’ cognitive reactions

**DOI:** 10.1186/s12912-025-04050-4

**Published:** 2025-11-21

**Authors:** Takaki Fukumori, Yuki Shirai, Yoshitake Takebayashi, Mariko Asai

**Affiliations:** 1 Graduate School of Technology, Industrial and Social Sciences, Tokushima University, 1–1, Minamijosanjima, Tokushima, 770-8502 Japan; 2 Graduate School of Nursing, Kansai Medical University, Osaka, Japan; 3 Department of Health Risk Communication, Fukushima Medical University School of Medicine, Fukushima, Japan; 4 Faculty of Pharma-Sciences, Teikyo University, Tokyo, Japan

**Keywords:** Compassion fatigue, Cancer, Nurses, Secondary traumatic stress, Traumatic events, Cognitive reactions

## Abstract

**Background:**

Continuous interaction with patients with cancer is key in the onset of compassion fatigue (CF) among nurses, and cognitive reactions are hypothesized to mediate the relationship between patient interactions and CF. This study develops and evaluates a model addressing traumatic events in patients with cancer, nurses’ cognitive reactions, and professional quality of life, including CF.

**Methods:**

A cross-sectional survey was conducted among nurses working at designated cancer hospitals. The Professional Quality of Life Scale was used to measure CF, while items assessing patient traumatic events and nurses’ cognitive reactions were developed based on previous qualitative findings. Structural equation modeling was employed to examine associations among variables.

**Results:**

Data from 536 participants were analyzed. Among five event categories, “bad news from doctors” directly affected CF; the others indirectly affected CF, mediated by four subfactors of cognitive reactions: “reconsideration of the meaning of life,” “desire to avoid one’s professional duties,” “sense of professional mission,” and “compassion for patients and their families.” All effects on CF, except “compassion for patients and their families” (*β*=-0.357, *p* = 0.001), were positive.

**Conclusions:**

These findings highlight the significance of specific traumatic events experienced by patients and cognitive reactions to these events at the onset of CF among cancer care nurses. Preventing CF is conceivable by targeting nurses’ cognitive reactions—especially thoughts confronting life’s meaning, desire to avoid or escape professional duties, and a sense of mission as a nurse—while promoting compassionate thoughts toward patients and their families.

**Supplementary Information:**

The online version contains supplementary material available at 10.1186/s12912-025-04050-4.

## Introduction

Nurses provide complex daily care to patients with various conditions, particularly those working with patients with cancer. It is well known that cancer care nurses experience significant stress [1, 2], similar to that encountered by the patients and their families. A notable example is the phenomenon of “compassion fatigue” (CF), which reflects natural consequent behaviors and emotions resulting from contact with a traumatic event experienced by a significant other (e.g., a patient). It arises from wanting to help that significant other and is synonymous with secondary traumatic stress (STS) [3–5]. Ongoing contact and interaction with patients are essential for their development [5]. A recent systematic review reported that 22% of nurses working in cancer care experienced a high prevalence of CF (STS) [6]. CF not only affects nurses’ well-being, which encompasses their state of being physically, mentally, and socially well and fulfilled, but also compromises the quality of the care they provide to patients [7–10].

To develop effective strategies to support cancer care nurses who perform such demanding duties, it is crucial to understand the mechanisms underlying CF development more deeply. The significance of cognitive variables in CF occurrence has been increasingly emphasized. For instance, CF has been linked to the disruption of caregivers’ schemas [4, 11], as well as changes in beliefs, expectations, and assumptions, which are also recognized as indicators of CF [12]. A scoping review further suggested that nurses’ personal beliefs regarding the nursing care they provide can contribute significantly to CF [13]. Moreover, self-evaluation has been identified as a mediating factor of CF development [14]. Collectively, these findings indicate that cognitive variables mediate the relationship between exposure to patient traumatic events and CF, highlighting the importance of addressing caregivers’ cognitive factors for both prevention and recovery. Despite these insights, the empirical models that integrate specific cognitive variables involved in CF require further exploration [15]. The relationship between the methods involved in the cognitive processing of trauma and CF (STS) has been gradually examined [16–18]; however, the exploration of the specific content of cognitive factors contributing to CF development remains insufficient. Building on this background, Fukumori et al. [19, 20] identified the categories of cognitive factors that contribute to CF and their triggering events through qualitative research. However, these studies did not empirically examine the relationships among the said factors or their impact on CF, thus highlighting the need for further quantitative analyses.

This study aims to quantitatively examine the categories derived from previous qualitative studies and elucidate the onset process of CF in cancer care nurses, focusing specifically on cognitive factors. Drawing on Hofmann’s [21] cognitive-behavioral model, this study constructs a framework that outlines the process as follows: triggers (traumatic event of the patient with cancer), state cognitions (nurses’ cognitive reactions to the event), and subjective experiences/physiological symptoms/behavioral responses (CF). Furthermore, we employ the concept of professional quality of life (QOL) [22], which refers to the overall quality of caregiving roles, including both positive and negative aspects of one’s work [23]; it also encompasses not only CF but also burnout (BO) and compassion satisfaction (CS), defined as the professional fulfillment derived from helping others and effectively performing one’s duties. This model provides a comprehensive understanding of the unique aspects of the CF process. By empirically presenting such a model, nurses can gain a deeper understanding of CF according to their specific situations and circumstances. This understanding could facilitate the development of effective, individualized cognitive interventions, ultimately enhancing both the well-being of nurses and the quality of patient care in oncology.

## Methods

### Design, participants, and setting

A cross-sectional survey was conducted with nurses working at designated cancer hospitals in three different regions across Japan from October 2020 to February 2021. A total of 1,018 questionnaires were distributed, and the survey was conducted anonymously. Participation in the survey was voluntary, and informed consent was obtained through participants’ confirmation on the questionnaire’s face sheet. The survey was conducted using a two-week placement method.

## Measures

### Data were collected using the following measures

#### Demographic variables

Demographic data, including age, sex, and academic history, were collected. Similarly, professional context data were gathered, namely on years of post-licensure nursing experience, years of experience in cancer care, working hours per month, and department mainly engaged in. Because the survey was conducted during the COVID-19 pandemic, nursing and end-of-life care experience with people infected with COVID-19 were also included as survey items, considering their potential influence on professional QOL.

#### Traumatic events in patients with cancer/nurses’ cognitive reactions

We developed items measuring the frequency of contact with patients with cancer who experienced traumatic events and the frequency of occurrence of cognitive reactions while interacting with these patients over the past three months. The procedure for developing each group of items was as follows. First, the items representing traumatic events experienced by patients with cancer, as well as nurses’ cognitive reactions to these events, were developed based on findings from qualitative studies [19, 20]. Second, a hearing investigation with three expert nurses (two certified palliative care nurses and a certified oncology nurse specialist) was conducted to review the phrasing of each item and identify any additional essential items related to traumatic events, leading to the inclusion of two more items. Third, the items’ content validity was assessed by four nurses, including the three aforementioned specialists and a general nurse. Ultimately, the list of traumatic events experienced by patients with cancer comprised 15 items, whereas the nurses’ cognitive reactions comprised 40 items. All items were rated on a 5-point Likert scale ranging from 0 (never) to 4 (very often).

#### Professional QOL

Professional QOL, including CF, was measured using the ProQOL Scale for Japanese Nurses (ProQOL-JN) [24]—a Japanese version of ProQOL-5 [23]. It comprises three subscales: “CF,” “CS,” and “BO.” The three subscales comprised 10 items each, and all items were rated on a 5-point Likert scale ranging from 1 (never) to 5 (very often). The ProQOL-JN has been previously validated in Japanese nurses and demonstrated reliability and validity [24].

### Statistical analyses

All statistical analyses were performed using R software (version 4.2.3). The item-level missing data rates were 1.1% (range: 0.6–1.9%) for nurses’ cognitive reactions, 0.5% (range: 0.2–0.8%) for the ProQOL-JN, and 0.7% (range: 0.2–1.5%) for the full model (comprising nurses’ cognitive reactions, the ProQOL-JN, and traumatic events in patients). This indicated a limited amount of missing data with minor item-level variation. As the Weighted Least Squares Mean and Variance adjusted (WLSMV) estimator does not support imputation methods such as Full-Information Maximum Likelihood, listwise deletion was applied. The resulting analytic sample sizes were 513 for nurses’ cognitive reactions, 525 for the ProQOL-JN, and 488 for the full model. For traumatic events, the mean of a group of items theoretically defined in a study [20] was calculated and treated as an observed variable. Additional items from the expert survey were then assigned to existing or new categories after discussions among the coauthors.

Structural equation modeling (SEM) was conducted in three sequential steps to examine the mediation pathways. First, the measurement model for cognitive reactions was validated to ensure the adequacy of the factor structure. Second, the measurement model for the ProQOL-JN was validated by employing the top three loading items from each of the three factors identified in the previous factor analyses. Finally, a mediation model was tested to assess how traumatic events experienced by patients with cancer influenced professional QOL through cognitive reactions.

To account for the indicators’ ordinal nature, the WLSMV estimation was employed for both the factor analyses and SEM. Model fit was evaluated using the comparative fit index (CFI), root mean square error of approximation (RMSEA), and standardized root mean square residual (SRMR), with the following cut-off criteria: CFI ≥ 0.95; RMSEA < 0.07 for acceptable fit and < 0.06 for good fit; SRMR < 0.08 for acceptable fit and < 0.05 for good fit [25].

To control for the potential influence of nurses’ experience with people infected with COVID-19, this variable was included as a covariate in the model. During the early phase of the pandemic, contemporaneous reports noted that the constant use of personal protective equipment, visitor restrictions, frequent staff reassignments, heightened fear of infection, and an increase in ethically complex situations increased nurses’ emotional labor and could exacerbate compassion fatigue [26, 27]. Specifically, the paths from this covariate to the latent variables of cognitive reactions and professional QOL were specified. Participants who reported having either nursing or end-of-life care experience with people with COVID-19 were classified as having relevant nursing experience.

## Results

### Participant characteristics

In total, 536 nurses (52.7%) participated in the research. Table 1 summarizes their demographic and clinical information. The mean age was 37.40 years (*SD* = 10.87; age range, 21–62 years), and 94.8% of the participants were women. Mean nursing and cancer nursing experiences were 14.02 (*SD* = 10.35) and 10.77 (*SD* = 8.83), respectively. A total of 87.7% of the participants were engaged in the departments of surgery, internal medicine, or a combination of both. Participants who responded “yes” to either nursing or end-of-life care with people with COVID-19 accounted for 30.0%.

**Table 1:** Participant characteristics (*N* = 536)

Characteristic	Mean, SD / *n*, %	
Age	37.40	10.87
Education		
Vocational school	277	51.7
Junior college	37	6.9
Undergraduate	193	36.0
Master’s program	12	2.2
Doctoral program	3	0.6
Others	7	1.3
Missing	7	1.3
Years of post-licensure nursing experience	14.02	10.35
Years of experience in cancer care	10.77	8.83
Working hours in a month	175.79	145.70
Department mainly engaged		
Surgical	202	37.7
Internal medicine	171	31.9
Mixed surgical and internal medicine	97	18.1
Palliative care	16	3.0
Others	30	5.6
Missing	20	3.7
Nursing experience with people infected with COVID-19		
Yes	114	21.3
No	420	78.4
Missing	2	0.4
End-of-life experience with people infected with COVID-19		
Yes	97	18.1
No	435	81.2
Missing	4	0.7

*SD*: standard deviation

Missing data: 1.7% age (*n* = 9), 0.7% years of post-licensure nursing experience (*n* = 4), 1.1% years of experience in cancer care (*n* = 6), 4.5% working hours in a month (*n* = 24)

### Categorization of traumatic events in patients with cancer

Regarding traumatic events, based on the theoretical framework established in previous research [20], a list of 15 items was categorized through discussions among three clinical experts (the first, second, and fourth authors), including clinical psychologists and nurses, all of whom hold PhDs. The categorization was refined to reflect professional expertise and ensure alignment with the clinical context. The categories of traumatic events experienced by patients were as follows: “worsening of physical condition,” referring to the deterioration of a patient’s medical condition; “bad news from doctors,” referring to the experience of receiving unfavorable news about a diagnosis or medical condition; “difficulty in treatment,” referring to situations where treatment does not progress as expected; “emotional conflict with family,” referring to difficulties in smoothly communicating intentions or wishes with family members; and “loss of personal autonomy,” referring to the inability to live one’s own life in one’s own way.

### Factor analysis of nurses’ cognitive reactions

Guided by parallel analysis, we conducted an exploratory factor analysis (EFA; minimum residual extraction) with promax rotation. The full standardized pattern matrix is provided in Supplementary Material, Table S1. Items with primary loadings < 0.50 were removed; however, when a factor contained fewer than three items with loadings ≥ 0.50, the loading threshold for that factor was relaxed to 0.30 to retain items for interpretability. The resulting seven-factor structure was then evaluated via confirmatory factor analysis (CFA), which showed acceptable fit (CFI = 0.937, RMSEA = 0.077, SRMR = 0.066). The seven factors were named based on the theoretical framework of previous research [19], as follows: “sense of professional inadequacy,” referring to the thought that one is not performing adequately as a professional; “reconsideration of the meaning of life,” referring to the thought of reflecting on one’s life and the meaning of living; “dissatisfaction with doctors,” referring to the thought of dissatisfaction with doctors; “immersion in patients,” referring to the thought of becoming empathically but excessively involved with patients, potentially including a tendency toward a self-centered stance; “desire to avoid one’s professional duties,” referring to the thought of wanting to escape or avoid work and patients; “sense of professional mission,” referring to the thought of pushing oneself owing to a strong sense of professional duty; and “compassion for patients and their families,” referring to the consideration of care and compassion toward patients and their families, involving an attempt to imaginatively understand their perspectives, including an imaginative form of identification with them (Table 2).

**Table 2:** Factor structure of nurses’ cognitive reactions

Items		α	Factor loadings
Sense of professional inadequacy		0.80	
1	Other nurses might have done a better job than me.		0.65
15	I am not being helpful to the patient.		0.75
37	I am causing distress to the patient due to my inability to handle situations well.		0.88
Reconsideration of the meaning of life		0.79	
30	What does it mean to die?		0.69
38	What would happen if I or my family were in the same situation as the patient?		0.77
31	While the patient is fighting illnesses, is it okay for me to live happily?		0.80
Dissatisfaction with doctors		0.92	
12	I wish doctors would communicate with the patient more empathetically.		0.93
24	Doctors should consider the patient more thoroughly during treatment.		0.88
5	I wish doctors would cooperate more with us in patient treatment.		0.86
Immersion in patients		0.78	
35	I do not want others to notice that I am getting emotionally involved with the patient.		0.90
25	Things would work out better if done my way for the patient and their surroundings.		0.65
36	Colleagues should be more empathetic in their approach to the patient.		0.66
Desire to avoid one’s professional duties		0.75	
11	I want to distance myself from the patient, even temporarily.		0.67
39	I want to step away from nursing as a profession.		0.65
20	I do not want to see the patient’s condition deteriorate.		0.81
Sense of professional mission		0.83	
33	I have to be helpful to the patient as a professional.		0.89
34	Taking responsibility for the patient is my duty.		0.79
40	I want to support the patient and their family to the best of my ability.		0.69
Compassion for patients and their families		0.87	
8	The patient’s sense of purpose in life has been taken away by the illness.		0.78
21	The patient must be going through a difficult time.		0.77
2	Will those left behind be okay when the patient is gone?		0.69
10	Is the patient’s mental and physical state okay?		0.74
13	If I were in the same position as the patient or their family, I would surely be tough.		0.79

### Model examination using SEM

Each variable was scored by calculating the mean values of the items within the subscales. For the ProQOL-JN, although CFA was performed using all items, model fit was poor. Therefore, the top three items for each factor were selected and used based on factor loadings from previous research [24]. Consequently, the model fit improved (CFI = 0.99, RMSEA = 0.08, SRMR = 0.05), and these items were subsequently adopted for further analysis.

**Fig. 1:**
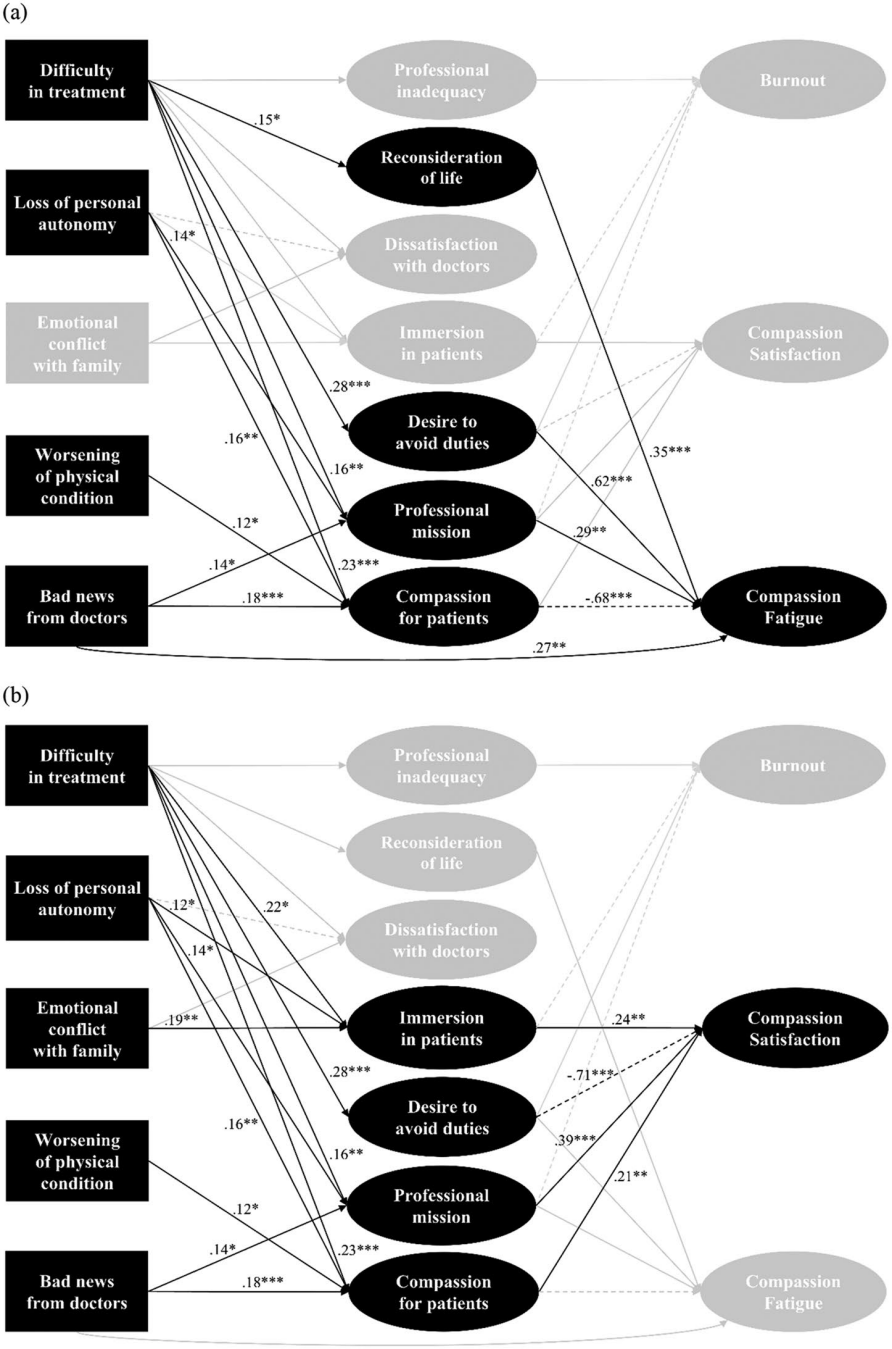

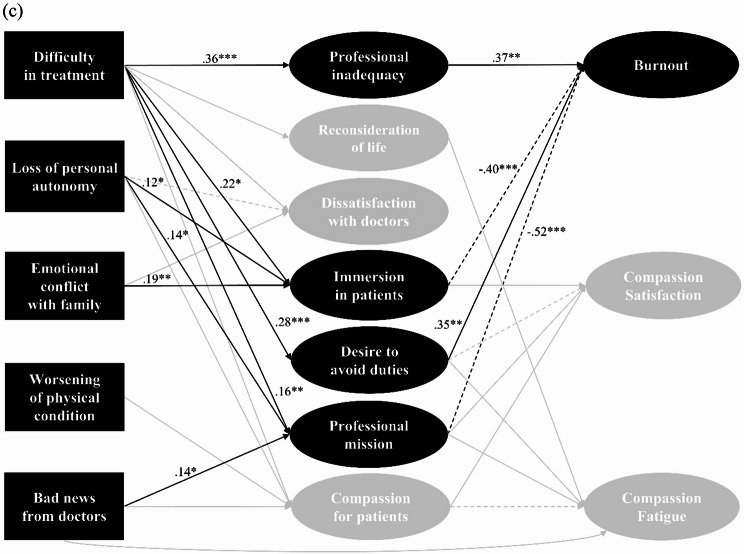
Standardized parameter estimates of the structural equation model. Three diagrams were created to highlight the relevant paths for each ProQOL-JN subscale (**a**: compassion fatigue, **b**: compassion satisfaction, **c**: burnout), because the entire path diagram was too complex. For clarity, standard errors are not displayed. ***p < 0.001, **p < 0.01, *p < 0.05

Figure 1 presents the model with only significant (*p* < 0.05) regression paths, while the covariances between the factors included in the cognitive reactions were omitted. Table 3 presents the descriptive statistics and the correlation matrix of the variables included in the model. In the model, the presence or absence of nursing experience with people with COVID-19 was incorporated as a covariate, with paths drawn to the latent variables of cognitive reactions and professional QOL. However, no significant effect of nursing experience with people with COVID-19 was found (*β* = 0.01–0.07, *p* = 0.126–0.899). The model’s goodness-of-fit indices were generally acceptable (CFI = 0.921, RMSEA = 0.057, SRMR = 0.075), though the CFI was slightly below the conventional threshold.

**Table 3:** Means and correlations among the variables included in the model

		Mean	SD	1	2	3	4	5	6	7	8	9	10	11	12	13	14
ProQOL-JN																	
1	Compassion fatigue	1.83	0.74	―													
2	Compassion satisfaction	3.08	0.79	− 0.17^***^	―												
3	Burnout	3.29	0.73	0.11^*^	− 0.70^***^	―											
Patient traumatic events																	
4	Worsening of physical condition	2.37	1.09	0.19^***^	0.04	− 0.10^*^	―										
5	Bad news from doctors	1.65	0.98	0.27^***^	0.06	− 0.10^*^	0.61^***^	―									
6	Difficulty in treatment	1.87	0.81	0.27^***^	0.02	− 0.08	0.63^***^	0.64^***^	―								
7	Emotional conflict with family	1.31	1.02	0.27^***^	0.01	− 0.10^*^	0.41^***^	0.45^***^	0.52^***^	―							
8	Loss of personal autonomy	2.11	1.04	0.24^***^	0.10^*^	− 0.11^*^	0.68^***^	0.60^***^	0.65^***^	0.44^***^	―						
Nurses’ cognitive reactions																	
9	Professional inadequacy	1.33	0.84	0.41^***^	− 0.14^**^	0.15^***^	0.27^***^	0.29^***^	0.37^***^	0.20^***^	0.35^***^	―					
10	Reconsideration of life	1.34	0.94	0.38^***^	0.02	− 0.05	0.25^***^	0.25^***^	0.28^***^	0.16^***^	0.29^***^	0.53^***^	―				
11	Dissatisfaction with doctors	1.73	1.13	0.27^***^	− 0.01	− 0.05	0.24^***^	0.28^***^	0.41^***^	0.36^***^	0.23^***^	0.29^***^	0.19^***^	―			
12	Immersion in patients	0.57	0.68	0.38^***^	0.04	− 0.12^**^	0.21^***^	0.29^***^	0.32^***^	0.30^***^	0.26^***^	0.40^***^	0.36^***^	0.35^***^	―		
13	Desire to avoid duties	1.32	0.93	0.51^***^	− 0.31^***^	0.22^***^	0.22^***^	0.25^***^	0.31^***^	0.22^***^	0.27^***^	0.57^***^	0.44^***^	0.31^***^	0.35^***^	―	
14	Professional mission	2.43	0.89	0.21^***^	0.33^***^	− 0.30^***^	0.32^***^	0.33^***^	0.33^***^	0.16^***^	0.34^***^	0.38^***^	0.38^***^	0.17^***^	0.29^***^	0.20^***^	―
15	Compassion for patients	2.46	0.81	0.22^***^	0.17^***^	− 0.14^**^	0.44^***^	0.47^***^	0.47^***^	0.26^***^	0.54^***^	0.51^***^	0.54^***^	0.23^***^	0.27^***^	0.39^***^	0.57^***^

The values presented in the table are Pearson correlation coefficients (*r*)

*SD*: standard deviation; ProQOL-JN: Professional Quality of Life Scale for Japanese Nurses. ***p < 0.001, **p < 0.01, *p < 0.05

All patient traumatic events, except for “emotional conflict with family,” were significantly associated with CF, either directly or indirectly. The cognitive reactions mediating the relationship between these events and CF were identified as “reconsideration of the meaning of life,” “desire to avoid one’s professional duties,” “sense of professional mission,” and “compassion for patients and their families.” Distinctive pathways were identified in relation to CS and BO. Specifically, “reconsideration of the meaning of life” was associated only with CF (*β* = 0.35, *p* < 0.001), while “immersion in patients” showed no association with CF but demonstrated a negative association with BO (*β*=-0.40, *p* < 0.001) and a positive association with CS (*β* = 0.24, *p* = 0.001). The cognitive reaction “sense of professional mission” was found to promote both CF (*β* = 0.29, *p* = 0.004) and CS (*β* = 0.39, *p* < 0.001), whereas “sense of professional inadequacy” was exclusively associated with BO (*β* = 0.37, *p* = 0.001). Furthermore, “bad news from doctors” directly influenced CF (*β* = 0.27, *p* = 0.003) but exerted a suppressive effect on CF when mediated by “compassion for patients and their families.”

## Discussion

This study aimed to quantitatively validate categories derived from qualitative research and propose a model to elucidate the process by which exposure to patient traumatic events leads to the onset of CF in cancer care nurses through their cognitive reactions. Factor analysis indicated that the cognitive reactions identified through the qualitative research could be understood using a seven-factor solution. A significant weak-to-moderate positive correlation at the 0.1% level was observed between all subscales of patient traumatic events, nurses’ cognitive reactions, and CF. The items regarding patient events and nurses’ cognitive reactions were developed based on findings from prior qualitative studies aimed at identifying cognitive factors related to the onset of CF [19, 20]; the results suggested criterion-related validity for these item groups. The results of the SEM using these variables further demonstrated that some traumatic events experienced by patients predicted CF, and that this effect was mediated by nurses’ cognitive reactions. This finding adds empirical support to the literature by highlighting the importance of cognitive factors in the development of CF.

Although not focused on nurses, a study involving oncologists [28] found that “subjective experience” plays a more crucial role in levels of CF than the “reporting of events.” As discussed above, although medical staff cannot alter their exposure to patient suffering and death, they can change their perceptions of their roles and experiences. Therefore, our findings underscore the significance of developing intervention methods that focus on nurses’ cognitive reactions.

In the SEM analysis, comparison of the paths to CF with those to CS and BO revealed certain unique findings regarding CF. First, “reconsideration of the meaning of life” was found to be associated only with CF. Medical staff frequently encounter existential questions and engage in reflections on the meaning of life while caring for patients with serious illnesses such as cancer and/or dying patients [29]. However, research on hospice nurses has indicated a distinction in the psychological impact of meaning-related processes: the presence of meaning in life is associated with lower psychological distress and negative affect, whereas the search for meaning in life is correlated with higher negative affect [30]. The present findings align with those of the aforementioned study [30], suggesting that, in the existentially demanding environment of cancer care, reconsidering and actively seeking meaning in life may increase vulnerability to stressors in the workplace. It may also induce guilt over privileged circumstances compared to those of patients [19]. Such responses could reflect a blurring of boundaries between the lives of patients and medical staff, warranting the need for caution when existential questions arise, related to, for example, “reconsideration of the meaning of life.” Concurrently, whether such reconsideration leads to vulnerability may depend on the nature of one’s spirituality. Recent studies suggest that spiritual well-being, a subjective state in which spirituality provides a sense of fulfillment and inner peace in everyday life, may buffer CF [31, 32]. One possible mechanism is that when spirituality is functioning well, the process of searching for meaning in life is supported by a spiritual framework and is therefore more likely to lead to acceptance and a sense of fulfillment. Conversely, when spirituality is not functioning, weaker connections to personal values and purpose can foster circular thinking and confine the search in repetitive thought patterns, which may be associated with higher levels of CF. Accordingly, when interpreting the relationship between “reconsideration of the meaning of life” and CF, both the level and quality of spirituality should be considered. Clinically, for nurses with heightened “reconsideration of the meaning of life,” meaning-centered and existential approaches (e.g., Meaning-Centered Psychotherapy [33]), which support values clarification and adaptive meaning-making may help buffer CF by providing a safe and structured context for meaning-making and self-reflection.

Second, “immersion in patients” was not associated with CF but demonstrated a negative association with BO and a positive association with CS. BO is generally considered more closely related to occupational factors such as workload, autonomy, and sense of accomplishment, rather than interpersonal relationships [34]. Additionally, as one of BO’s key characteristics is depersonalization, it is not surprising that a negative correlation with “immersion in patients” was observed. Conversely, the lack of association with CF may be explained by the items that constitute “immersion in patients,” including concealing one’s feelings from others and the desire to clarify and assert one’s own intentions. These elements reflect the tendency of maintaining clear boundaries with others. This tendency may explain why “immersion in patients” was not associated with CF, given that blurred boundaries in caregiving roles are known to contribute to CF [7]. Such an observation provides valuable insights into distinguishing between CF and CS.

Third, a “sense of professional mission” was found to enhance both the CF and CS scores. This finding is noteworthy because CS is typically conceptualized as a counterpoint to CF. While a sense of mission can enhance nurses’ motivation and joy in their work, it may also lead to self-imposed pressure. The key issue is the path that this sense of mission follows. Excessive drive rooted in a sense of mission may lead some nurses to give away their private time or engage in self-sacrifice. In this regard, our findings are consistent with previous research showing that self-sacrifice needs constitute a risk factor for CF [35], suggesting that this association may be generalizable across cultural contexts. Particularly in Japanese healthcare settings, workplace norms that valorize devotion and endurance are reportedly strong [36]; such an organizational climate may intensify the link between a sense of mission and CF by increasing the likelihood of a mission orientation translating into self-sacrifice. The critical takeaway is that, when cognitive reactions related to sense of mission arise, identifying them early and channeling them toward self-encouragement rather than self-sacrifice is essential. The present findings imply that such a redirection is achievable.

Finally, our finding that “compassion for patients and their families” mediates and suppresses the direct path from “bad news from doctors” to CF suggests that cognitive reactions related to compassion for patients may serve as a protective factor against CF. Conceptually, empathy involves sharing and understanding others’ emotions and, when directed toward their suffering, may elicit personal distress. By contrast, compassion denotes an other-oriented prosocial motivation to alleviate suffering and is associated with warmth and positive affect [37]. This distinction aligns with neuroscientific findings indicating that, while empathy alone can lead to distress, fostering compassion promotes positive affect [37, 38]. Consistent with this, compassion-based interventions have demonstrated benefits for overcoming distress among health-care professionals and in broader samples, suggesting clinical meaning to strengthen this protective factor [39, 40]. These results provide new insights into the ongoing debate concerning whether compassion can cause CF.

### Study limitations

This study has some limitations that should be addressed in future research. First, there was a potential sampling bias. Although efforts were made to diversify the regions and facilities sampled across Japan, the sample was ultimately drawn from large general hospitals, and approximately 95% of participants were women. Consequently, these findings may not be generalizable to male nurses or to nurses working in other settings, including smaller clinics or hospices. Future studies should incorporate a broader range of healthcare settings and a more gender-balanced sample to improve generalizability of the results. Second, the model fit was relatively modest, suggesting the need for further refinement. To improve the model’s robustness, additional studies using different samples are recommended, as well as efforts to refine the model further based on these findings. Finally, because the results were based on cross-sectional data, causality could not be established. Future research should incorporate longitudinal data to better assess the potential causal relationships among the variables examined.

### Implications

The findings of this study suggest the importance of examining interventions targeting specific cognitive factors among oncology nurses to either prevent or aid recovery from CF, because several cognitive factors are potentially significant obstacles in this setting. Reflecting on one’s work and personal life is crucial for preventing and recovering from CF [41, 42]. This study provides clearer guidance on “which perspectives” might be beneficial for such reflection. By equipping nurses with targeted tools for managing these responses, healthcare systems can support their staff’s well-being, ultimately fostering an environment in which sustainable and compassionate care for patients with cancer is prioritized. These insights are expected to guide future efforts in establishing supportive frameworks for healthcare professionals at risk of CF.

## Conclusion

This study suggests that specific “traumatic events experienced by patients with cancer” and “nurses’ cognitive reactions to these events” contribute to the onset of CF among nurses in cancer care. The results indicate that it may be possible to effectively prevent CF by targeting nurses’ cognitive reactions, particularly thoughts that confront the meaning of their own lives, the desire to avoid or escape professional duties, and a sense of mission as a nurse; compassionate consideration for patients and their families should also be promoted. However, as these results are based on cross-sectional data, further studies using longitudinal data are required to establish a causal relationship.

## Supplementary Information

Below is the link to the electronic supplementary material.

Supplementary Material 1

## Data Availability

The datasets generated and analyzed during the current study are not publicly available due to a lack of explicit participant consent for public data sharing; however, they are available from the corresponding author upon reasonable request.
